# 1-Methyl-1,2,3,4-tetrahydroisoquinoline, an Endogenous Neuroprotectant and MAO Inhibitor with Antidepressant-Like Properties in the Rat

**DOI:** 10.1007/s12640-013-9425-0

**Published:** 2013-09-25

**Authors:** Agnieszka Wąsik, Edyta Możdżeń, Jerzy Michaluk, Irena Romańska, Lucyna Antkiewicz-Michaluk

**Affiliations:** Department of Neurochemistry, Institute of Pharmacology, Polish Academy of Sciences, 12 Smetna Street, 31-343 Kraków, Poland

**Keywords:** 1-Methyl-1,2,3,4-tetrahydroisoquinoline, Rat forced swimming test, Desipramine, Depression, Metabolism of monoamines, MAO inhibitor

## Abstract

Oxidative stress is a major contributing factor in a range of brain pathologies and in the etiology of depression. 1-Methyl-1,2,3,4-tetrahydroisoquinoline (1MeTIQ) is an endogenous substance which is present in the mammalian brain and exhibits neuroprotective, and monoamine oxidase (MAO)-inhibiting properties. In the present study, in order to investigate the potential role of 1MeTIQ as an antidepressant, we tested antidepressant-like effects of 1MeTIQ in comparison with desipramine (a classic antidepressant) in the forced swimming test (FST), and using HPLC methodology, we measured the concentrations of monoamines (dopamine, noradrenaline, serotonin) and the rate of their metabolism. 1MeTIQ given alone as well as in combination with desipramine produced an antidepressant-like effect and decreased the immobility time in the FST. Neurochemical data have shown that 1MeTIQ like desipramine, activated the noradrenergic system. However, the mechanism of action of 1MeTIQ is broader than the actions of desipramine, and 1MeTIQ inhibits the MAO-dependent oxidation of dopamine and serotonin in all investigated structures. We can conclude that 1MeTIQ exhibits antidepressant-like activity in the FST in the rat. The mechanism of its antidepressant action differs from desipramine and seems to be mostly associated with the inhibition of the catabolism of monoamines and their increased concentrations in the brain. 1MeTIQ seems to be very beneficial from the clinical point of view as a reversible MAO inhibitor with a significant antidepressant effects.

## Introduction

One of the popular theories states that depression is caused by the depletion of monoaminergic activity (especially noradrenergic and/or serotoninergic). Additionally, different studies have suggested the role of dopamine in the pathogenesis of depression (Brown and Gershon 1993). There is a biochemical evidence indicating that dopaminergic system plays a role in the antidepressant-like effect in the forced swimming test (FST) (Renard et al. 2003). Desipramine is a classic tricyclic antidepressant (TCA) with a substantial selectivity for noradrenaline reuptake (Wroblewski et al. 1996) and via this mechanism it elevates the noradrenaline concentration. Desipramine has been studied extensively in behavioral models of depression in rodents (Antonio et al. 1988; Detke et al. 1995, 1997; Detke and Lucki 1996). Recent studies have suggested that oxidative stress may be involved in etiopathology of a variety of diseases, such as depression, anxiety, or cognitive dysfunctions (Bhattacharya and Muruganandam 2003). Preclinical and clinical experiments indicated that stress and depression led to reduction in the number of the adult hippocampal neurons (Tsankova et al. 2006). Some authors demonstrated that chronic exposure to stressful constraints in rodents produced depressive behavior (Berton et al. 2006; Kim and Han 2006; Covington et al. 2009; Kim et al. 2012). There is evidence that neuroprotective compounds possess antidepressant-like properties (Scapagnini et al. 2012). 1-Methyl-1,2,3,4-tetrahydroisoquinoline (1MeTIQ) is an endogenous compound showing neuroprotective (Antkiewicz-Michaluk et al. 2001, 2006) and antiaddictive (Wąsik et al. 2007) properties present in the mammalian brain (Yamakawa et al. 1999. Our earlier studies demonstrated that 1MeTIQ inhibited both monoamine oxidase A (MAO-A) and B (MAO-B) enzymatic activities and increased monoamine neurotransmitter levels in the brain (Patsenka and Antkiewicz-Michaluk 2004). 1MeTIQ inhibits the formation of 3,4-dihydroxyphenylacetic acid (DOPAC), lowers the production of free radicals and shifts dopamine catabolism toward the catechol-*O*-methyltransferase (COMT)-dependent *O*-methylation, and such mechanism of action seems to be important for its neuroprotective activity (Antkiewicz-Michaluk et al. 2001). The constant presence of 1MeTIQ in the mammalian brain suggests that 1MeTIQ may play a crucial physiological role as an endogenous regulator of dopaminergic activity (Vetulani et al. 2003). Additionally, as we showed earlier, 1MeTIQ administered systemically to rats produced a dose-dependent antidepressant-like effect in the forced swimming test (FST) (Wąsik et al. 2013). Monoamine oxidase inhibitors (MAOIs) are classic drugs in the treatment of depression. Already in the 1970s preclinical investigations demonstrated that MAOIs showed antidepressant-like properties (Porsolt et al. 1978). The FST described originally by Porsolt et al. (1977) is the most common test used for evaluation of antidepressant-like effects of different drugs. A relative rapidity in testing of antidepressant action and sensitivity to short-term antidepressant effects are the major advantages of the FST in drug discovery.

The aim of the present study was to investigate the antidepressant properties of 1MeTIQ given in a low dose alone and in combination with the classical antidepressant, desipramine in the FST. What is more, in the biochemical studies the rate of monoamine (dopamine, noradrenaline, and serotonin) metabolism was estimated in some rat brain structures. Additionally, in order to exclude the psychostimulating activity of 1MeTIQ, the locomotor activity of rats after administration of the investigated drugs was measured in actometers (Opto-Varimex activity monitors) linked on-line to an IBM-PC compatible computer.

## Materials and Methods

### Animals and Treatment

Behavioral tests were carried out on male Wistar rats of initial body weight 220–240 g (about 7 weeks old) kept under standard laboratory conditions, 6–8 to a large animal cage. All animals had free access to standard laboratory food and tap water and were maintained at room temperature (22 °C) under an artificial light/dark cycle (12/12 h, light on from 7 a.m.).

The rats were administered desipramine at a dose of 10 mg/kg intraperitoneally (i.p.) once 1 h before the forced swim test. 1MeTIQ in a dose of 10 mg/kg i.p. was administered once 15 min before desipramine injection. Control rats were treated with an appropriate solvent. Immediately after the end of behavioral experiments, the rats were killed by decapitation and different structures of the brain were dissected. The experiments were carried out between 9.00 and 16.00 h.

All procedures were carried out in accordance with the National Institutes of Health Guide for the Care and Use of Laboratory Animals and were granted an approval from the Bioethics Commission as compliant with Polish Law. All the experimental procedures were approved by the Local Bioethics Commission of the Institute of Pharmacology, Polish Academy of Sciences in Kraków.

### Drugs

1-Methyl-1,2,3,4-tetrahydroisoquinoline (1MeTIQ) was synthesized by Dr. Jan Boksa, (Department of Drug Chemistry, Institute of Pharmacology, Polish Academy of Sciences, Krakow, Poland). Purity of the compound was verified by measurement of the melting point, and homogeneity was assessed on a chromatographic column. Desipramine (Sigma-Aldrich, USA) and 1MeTIQ were dissolved in sterile 0.9 % NaCl solution and injected in a volume of 1 ml/kg.

### Behavioral Tests

#### Forced Swimming Test

The studies were carried out according to the method of Porsolt et al. (1978). The rats were placed in non-transparent plastic cylinders (height 50 cm, diameter 23 cm) containing 30 cm of water, maintained at 25 °C. Two swim sessions were conducted: an initial 15-min pretest followed 24 h later by a 5-min test. After both sessions, rats were removed from cylinders and returned to their home cages. Behavioral observations were performed according to Detke et al. (1995) and during the 5-min test session three different behaviors were recorded: immobility—when rats remained floating passively in the water; swimming—if they were making active swimming movements; climbing—when they were making active attempts in and out of the water with their forepaws, usually directed against the walls.

#### Locomotor Activity

The locomotor activity was examined in actometers (Opto-Varimex activity monitors, Columbus Inst., USA) linked on-line to an IBM-PC compatible computer. Each cage (43 × 44 × 25 cm) was surrounded with a 15 × 15 array of photocell beams located 3 cm above the floor surface as reported previously (Filip et al. 2007). Interruptions of these photocell beams were counted as a measure of horizontal locomotor activity defined as the distance traveled (in cm). Horizontal locomotor activity was recorded for 90 min and analyzed using Auto-Track Software Program (Columbus Instruments, USA). The rats received desipramine in a dose of 10 mg/kg i.p. acutely; 1MeTIQ was administered in a dose of 10 mg/kg i.p. also acutely. In the combination group, 1MeTIQ was given 15 min before desipramine administration. The control group was treated with saline. The animals were transferred to the experimental cages 15 min after desipramine injections. Horizontal locomotor activity was assessed for 90 min. Seven animals per group were used.

### Biochemical Assays

After the end of the behavioral experiments, the rats were killed by decapitation and the hypothalamus and striatum were dissected immediately and the obtained tissue was frozen on solid CO_2_ (−70 °C) and stored until used for biochemical assay. Dopamine and its metabolites, 3,4-dihydroxyphenylacetic acid (DOPAC), 3-methoxytyramine (3-MT) and final metabolite, homovanillic acid (HVA); serotonin (5-HT) and its metabolite 5-hydroxyindoleacetic acid (5-HIAA); noradrenaline and its metabolite 3-metoxy-4-hydroxyphenylglycol (NMN) were assayed by means of high-performance liquid chromatography (HPLC) with electrochemical detection. The chromatograph HP 1050; Hewlett-Packard, Golden, CO, USA was equipped with C18 columns. The tissue samples were weighed and homogenized in ice-cold 0.1 M perchloroacetic acid containing 0.05 mM ascorbic acid. After centrifugation (10,000×*g*, 5 min), the supernatants were filtered through RC 58 0.2-im cellulose membranes (Bioanalytical Systems, West Lafayette, IN, USA). The mobile phase consisted of 0.05 M citrate–phosphate buffer, pH 3.5, 0.1 mM EDTA, 1 mM sodium octyl sulfonate, and 3.5 % methanol. The flow rate was maintained at 1 ml/min. Dopamine, serotonin, noradrenaline and its metabolites were quantified by peak height comparison with standards run on the day of analysis.

### Statistical Analysis

The results of behavioral (FST) test was measured by means of a one-way analysis of variance (ANOVA) and neurochemical tests were analyzed by means of a two-way analysis of variance (ANOVA) followed when appropriate, by Duncan’s post hoc test. The results were considered statistically significant when *P* < 0.05. The data from locomotor activity test were analyzed by means of a two-way analysis of variance (ANOVA) for repeated measures, followed when appropriate, by Duncan’s post hoc test. The results were considered statistically significant when *P* < 0.05.

## Results

### The Effect of 1MeTIQ, Desipramine, and Their Combined Administration on Performances of Rats in the Forced Swimming Test

**Fig. 1:**
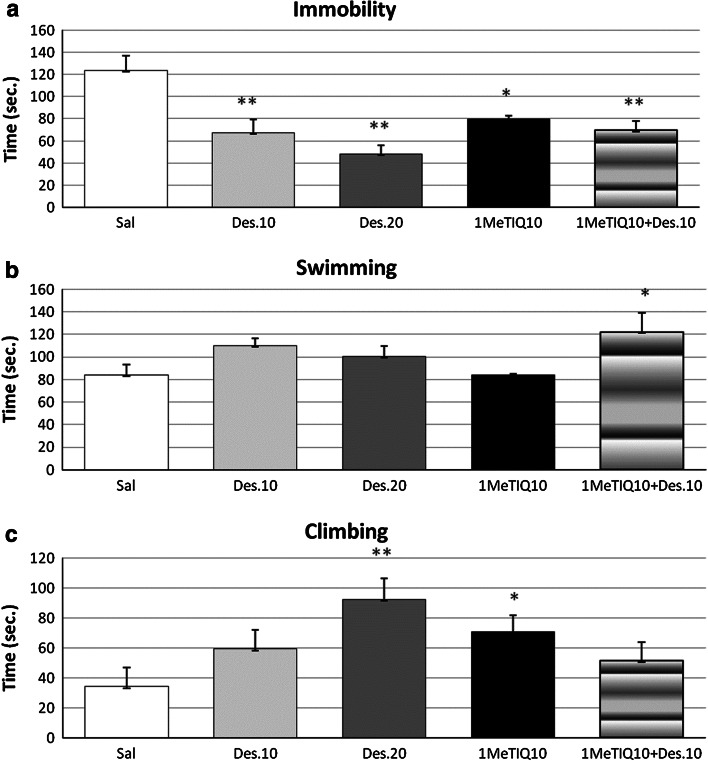
The effect of 1MeTIQ on desipramine-induced changes in the forced swimming test (FST). The rats received a single injection of saline (control), desipramine in two doses (10 and 20 mg/kg i.p.), or 1MeTIQ (10 mg/kg i.p.). In the mixed group, 1MeTIQ was given 15 min before desipramine (10 mg/kg i.p.) injection. The behavioral test (FST) was conducted 1 h after desipramine injection. The rats were placed into the cylinder for 5 min and during this time was measured three types of behavior: immobility (**a**), swimming (**b**), and climbing (**c**). The data are mean ± SEM, the number of animals was *n* = 6; * *P* < 0.05, ** *P* < 0.01 difference from control group (SAL) with Duncan test

Acute administration of both doses of desipramine (10 and 20 mg/kg i.p.) or 1MeTIQ (10 mg/kg i.p.) produced a significant decrease in the immobility time in the FST (*F*[4,22] = 8.93, *P* < 0.01) (Fig. 1a). The one-way ANOVA indicated also a significant reduction of the immobility time in the combination group, when 1MeTIQ (10 mg/kg i.p.) was combined with desipramine (10 mg/kg i.p.) (Fig. 1a) with simultaneous prolongation of the swimming time (*P* < 0.05) (Fig. 1b). The statistical analysis revealed a significant increase in the climbing activity after systemic administration of 1MeTIQ and desipramine in a higher dose of 20 mg/kg (*F*[4,22] = 3.36, *P* < 0.05) with no effect of desipramine in a dose of 10 mg/kg i.p. (Fig. 1c).

### The Effect of 1MeTIQ, Desipramine, and Their Combined Administration on the Locomotor Activity in Rats

**Fig. 2:**
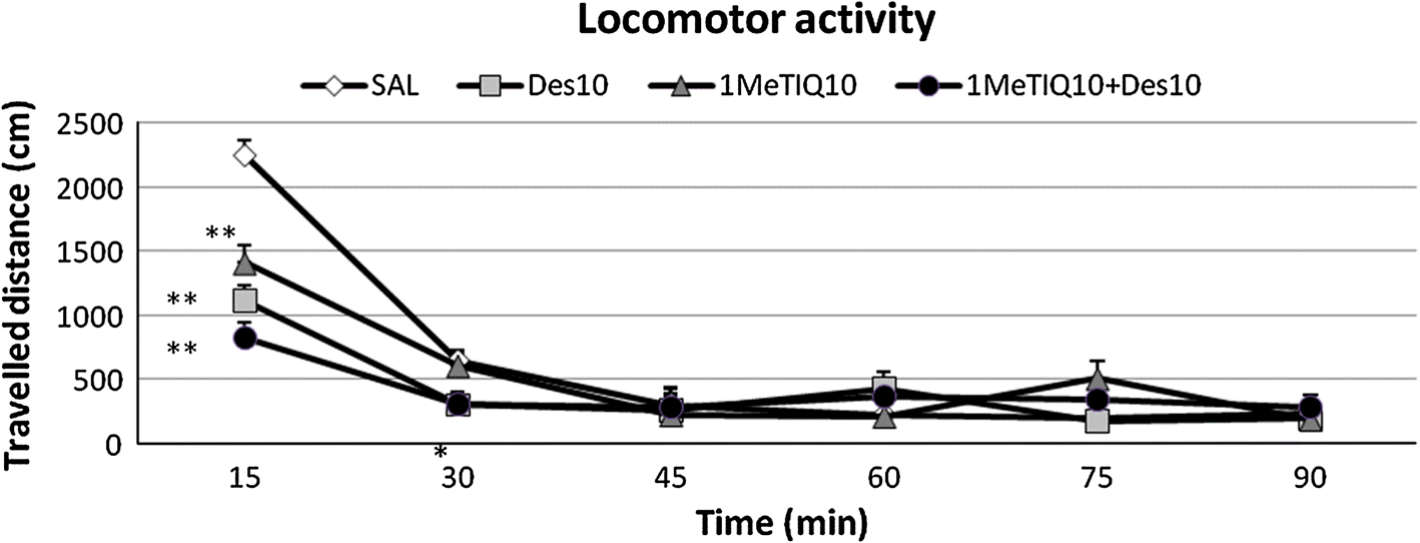
The influence of 1MeTIQ on desipramine-induced changes on the rats locomotor activity. The rats received a single injection of saline (control), desipramine (10 mg/kg i.p.), or 1MeTIQ (10 mg/kg i.p.). In the mixed group, 1MeTIQ was given 15 min before desipramine injection and 15 min later were placed into actometers for 90 min. The data are mean ± SEM, the number of animals was *n* = 7; The data were analyzed by means of two-way analysis of variance (ANOVA) for repeated measures, followed when appropriate, by Duncan’s post hoc test. Statistical significance: * *P* < 0.05, ** *P* < 0.01 versus control group

Figure 2 shows that acute administration of 1MeTIQ (10 mg/kg i.p.) or desipramine (10 mg/kg i.p.) produced a significant decrease in locomotor activity of rats (*P* < 0.01). Joint administration of 1MeTIQ and desipramine produced a stronger decrease in locomotor activity of rats. The two-way ANOVA for repeated measures demonstrated no effect of 1MeTIQ (10 mg/kg i.p.) (*F*[1,24] = 0.5, N.S.) or desipramine (10 mg/kg i.p.) (*F*[1,24] = 3.78, N.S.). Also interaction between 1METIQ versus desipramine was not significant (*F*[1,24] = 0.4, N.S.). At the same time, the two-way ANOVA for repeated measures indicated a significant effect of TIME (*F*[5,120] = 114.9, *P* < 0.01), significant effect of interaction of TIME versus 1MeTIQ (*F*[5,120] = 9.9, *P* < 0.01) and TIME versus desipramine (*F*[5,120] = 19.78, *P* < 0.01). The statistical analysis showed no effect of interaction of TIME versus 1MeTIQ versus desipramine (*F*[5,120] = 1.98, N.S.).

### Neurochemical Studies

#### The Influence of 1MeTIQ, Desipramine, and Their Combined Administration on the Concentration of Dopamine and Its Metabolites in the Rat Brain Structures

In the hypothalamus, a two-way ANOVA revealed a significant effect (*F*[1,19] = 24.9, *P* < 0.01) of treatment with 1MeTIQ on dopamine concentration (Table 1). The same statistical test demonstrated no effect of desipramine (*F*[1,19] = 0.62, N.S.) and no interaction between 1MeTIQ versus desipramine (*F*[1,19] = 3.39, N.S.) for the level of dopamine (Table 1). The post hoc test demonstrated that 1MeTIQ elevated dopamine concentration as well given alone as combined with desipramine (by about 20 and 40 %, respectively). The two-way ANOVA indicated a significant effect of 1MeTIQ (*F*[1,19] = 9.5, *P* < 0.01) but no significant effect of desipramine (*F*[1,19] = 0.48, N.S.) on the level of DOPAC. The statistical analysis showed no effect of 1MeTIQ versus desipramine interaction (*F*[1,19] = 3.5, N.S.) in influencing DOPAC concentration (Table 1). The post hoc Duncan’s test demonstrated that 1MeTIQ administration significantly reduced the level of DOPAC (by about 40 %, *P* < 0.01) in the hypothalamus. The two-way ANOVA revealed a significant effect of 1MeTIQ (*F*[1,19] = 12.1, *P* < 0.01) and no effect of desipramine (*F*[1,19] = 4.03, N.S.) on 3-MT concentration in the hypothalamus (Table 1). At the same time, the interaction between 1MeTIQ versus desipramine in influencing the level of 3-MT also was significant (*F*[1,19] = 8.3, *P* < 0.01). The post hoc test showed that 1MeTIQ increased the level of 3-MT by 200 % (*P* < 0.01). In the hypothalamus, the two-way ANOVA showed no effect of 1MeTIQ (*F*[1,19] = 0.01, N.S.) and no effect of desipramine (*F*[1,19] = 3.5, N.S.) on HVA concentration (Table 1). The same statistical analysis demonstrated no effect of 1MeTIQ versus desipramine interaction (*F*[1,19] = 0.09, N.S.) in modulating the level of HVA (Table 1).

**Table 1:** The influence of 1MeTIQ, desipramine, and combined administration on dopamine and its metabolites concentrations in rat brain structures

Treatment 1 (mg/kg)	Treatment 2 (mg/kg)	DA (ng/g t)	DOPAC (ng/g t)	3-MT (ng/g t)	HVA (ng/g t)
Hypothalamus					
Saline	Saline	456 ± 21	126 ± 10	2 ± 0.4	35 ± 6
	Desipramine 10	425 ± 22	101 ± 9	3 ± 0.4	28 ± 3
1MeTIQ 10	Saline	548 ± 43*^#^	78 ± 12**	6 ± 1.2**^##^	37 ± 7
	Desipramine 10	625 ± 30**^##^	89 ± 6*	3 ± 0.3	26 ± 3
Effect of 1MeTIQ		*F*_(1/19)_ = 24.9	*F*_(1/19)_ = 9.5	*F*_(1/19)_ = 12.1	*F*_(1/19)_ = 0.01
		*P* < 0.01	*P* < 0.01	*P* < 0.01	N.S.
Effect of desipramine		*F*_(1/19)_ = 0.62	*F*_(1/19)_ = 0.48	*F*_(1/19)_ = 4.03	*F*_(1/19)_ = 3.5
		N.S.	N.S.	N.S.	N.S.
Effect of interaction 1MeTIQ + desipramine		*F*_(1/19)_ = 3.39	*F*_(1/19)_ = 3.5	*F*_(1/19)_ = 8.3	*F*_(1/19)_ = 0.09
		N.S.	N.S.	*P* < 0.01	N.S.
Striatum					
Saline	Saline	11,404 ± 503	1,738 ± 107	306 ± 28	1,016 ± 98
	Desipramine 10	12,871 ± 453	1,853 ± 59	368 ± 24	1,094 ± 48
1MeTIQ 10	Saline	11,296 ± 656	1,253 ± 79**^##^	343 ± 15	1,234 ± 193
	Desipramine 10	13,083 ± 443*	1,473 ± 67^#^	325 ± 17	947 ± 57
Effect of 1MeTIQ		*F*_(1/19)_ = 0.92	*F*_(1/19)_ = 22.3	*F*_(1/19)_ = 0.01	*F*_(1/19)_ = 0.15
		N.S.	*P* < 0.01	N.S.	N.S.
Effect of desipramine		*F*_(1/19)_ = 9.26	*F*_(1/19)_ = 3.5	*F*_(1/19)_ = 0.74	*F*_(1/19)_ = 1.26
		*P* < 0.05	N.S.	N.S.	N.S.
Effect of interaction		*F*_(1/19)_ = 0.09	*F*_(1/19)_ = 3.3	*F*_(1/19)_ = 2.52	*F*_(1/19)_ = 3.85
1MeTIQ + desipramine		N.S.	N.S.	N.S.	N.S.

1MeTIQ was administered once in a dose 10 mg/kg i.p. Desipramine was administered in acute dose 10 mg/kg i.p. In the mixed group, 1MeTIQ was given 15 min before desipramine injection. Control group was treated with saline. The rats were decapitated about 1 h after desipramine administration, immediately after completion of a behavioral test (FST). The results are expressed as the mean ± SEM from 4 to 6 samples. The indices were calculated using concentrations from individual tissue samples. The concentration of dopamine and its metabolites were measured in ng/g tissue. The data were analyzed by means of two-way analysis of variance ANOVA, followed when appropriate, by Duncan’s post hoc test. Statistical significance: * *P* < 0.05, ** *P* < 0.01 versus Control group; ^#^ *P* < 0.05, ^##^ *P* < 0.01 versus desipramine-treated group

In the striatum, the two-way ANOVA indicated no effect of 1MeTIQ (*F*[1,19] = 0.92, N.S.) on dopamine concentration but the effect of desipramine (*F*[1,19] = 9.26, *P* < 0.01) was significant (Table 1). The same statistical test demonstrated no effect of 1MeTIQ versus desipramine interaction (*F*[1,19] = 0.09, N.S.) for the level of dopamine (Table 1). The two-way ANOVA revealed a significant effect of 1MeTIQ (*F*[1,19] = 22.3, *P* < 0.01) on the level of DOPAC. The statistical analysis showed no effect of desipramine (*F*[1,19] = 3.5, N.S.) or 1MeTIQ versus desipramine interaction (*F*[1,19] = 3.3, N.S.) in influencing DOPAC concentration (Table 1). The post hoc Duncan’s test demonstrated that 1MeTIQ administration significantly reduced the level of DOPAC (by about 30 %, *P* < 0.01) in the striatum. The two-way ANOVA showed no effect of treatment with 1MeTIQ (*F*[1,19] = 0.01, N.S.) and desipramine (*F*[1,19] = 0.74, N.S.) on the level of 3-MT. The same statistical test demonstrated no effect of 1MeTIQ versus desipramine interaction (*F*[1,19] = 2.52, N.S.) for 3-MT concentration (Table 1). The two-way ANOVA showed no effect of 1MeTIQ(*F*[1,19] = 0.15, N.S.) and no effect of desipramine (*F*[1,19] = 1.26, N.S.) on HVA concentration in the striatum (Table 1). The same statistical analysis demonstrated no effect of 1MeTIQ versus desipramine interaction (*F*[1,19] = 3.85, N.S.) in affecting the level of HVA (Table 1).

#### The Effect of 1MeTIQ, Desipramine, and Their Combined Administration on the Rate of Dopamine Metabolism in the Rat Hypothalamus

**Fig. 3:**
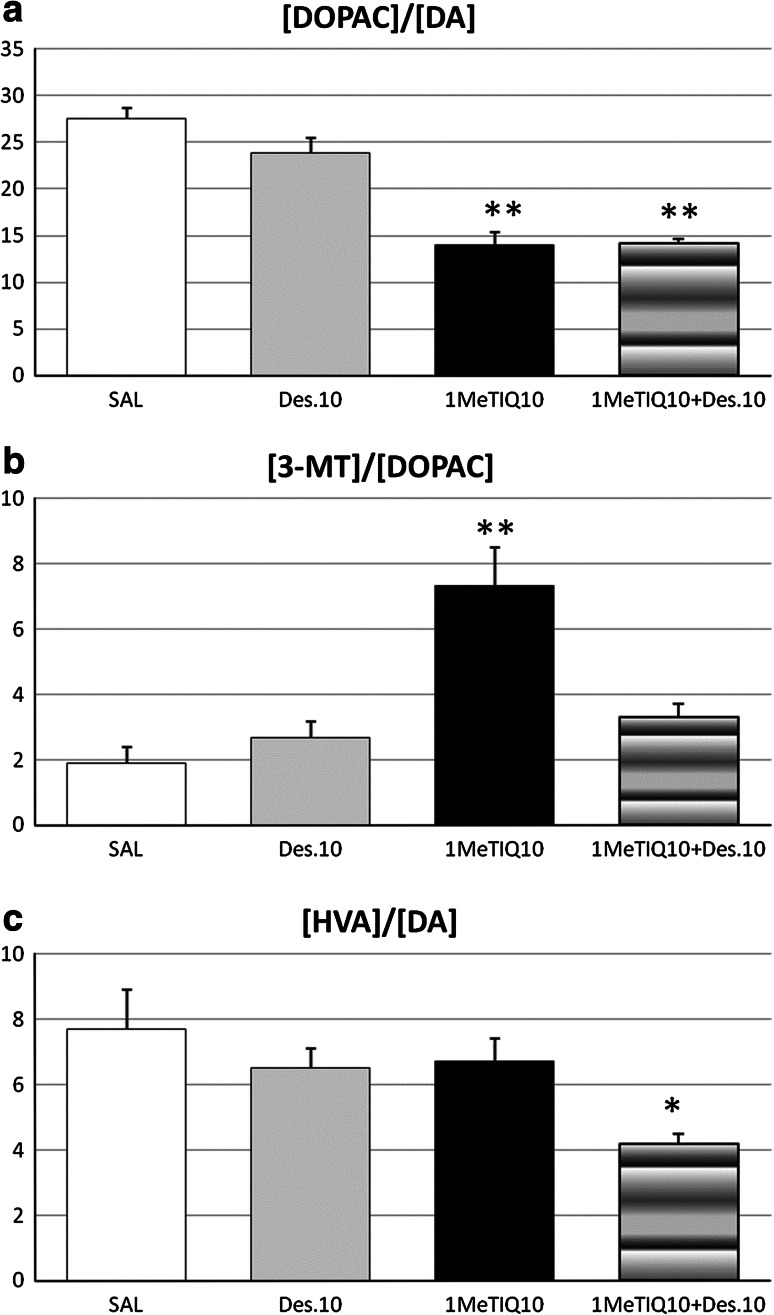
The effect of 1MeTIQ compared with desipramine as well as combined administration of both this drugs on the rate: of dopamine oxidation expressed as ([DOPAC]/[DA]) × 100 (**a**); on the inhibition of dopamine reuptake expressed as ([3-MT]/[DOPAC]) × 100 (**b**); and final dopamine metabolism expressed as ([HVA]/[DA]) × 100 (**c**) in the rat hypothalamus. The rats received a single injection of saline (control), desipramine (10 mg/kg i.p.) or 1MeTIQ (10 mg/kg i.p.). In the mixed group, 1MeTIQ was given 15 min before desipramine injection. The rats were decapitated about 1 h after desipramine administration, immediately after completion of a behavioral test (FST). The results are expressed as the mean ± SEM from 4 to 6 samples. The indices were calculated using concentrations from individual tissue samples. The data were analyzed by means of two-way analysis of variance ANOVA, followed when appropriate, by Duncan’s post hoc test. Statistical significance: * *P* < 0.05, ** *P* < 0.01 versus control group

In the hypothalamus, the two-way ANOVA revealed a significant effect of 1MeTIQ on the rate of dopamine oxidation pathway measured as the index [DOPAC]/[DA] × 100 (*F*[1,19] = 74.9, *P* < 0.01). At the same time, the effect of desipramine (*F*[1,19] = 1.77, N.S.) or 1MeTIQ versus desipramine interaction (*F*[1,19] = 2.23, N.S.) was not significant (Fig. 3a). The Duncan’s post hoc test indicated that 1MeTIQ (10 mg/kg i.p.) reduced the rate of dopamine oxidation pathway by about 45 % (*P* < 0.01) (Fig. 3a). The same statistical analysis demonstrated a significant effect of 1MeTIQ (*F*[1,19] = 24.7, *P* < 0.01) and desipramine (*F*[1,19] = 6.83, *P* < 0.05) on the reuptake of dopamine measured as the index [3-MT]/[DOPAC] × 100 (Fig. 3b). Also the interaction between 1MeTIQ and desipramine was significant (*F*[1,19] = 15.4, *P* < 0.01). The post hoc test showed that 1MeTIQ given alone strongly elevated the dopamine reuptake rate (by about 350 %, *P* < 0.01). The two-way ANOVA indicated no effect of 1MeTIQ (*F*[1,19] = 3.37, N.S.) or desipramine (*F*[1,19] = 4.32, N.S.) on the rate of dopamine catabolism (Fig. 3c). Also the interaction between 1MeTIQ and desipramine was not significant (*F*[1,19] = 0.46, N.S.).

#### The Influence of 1MeTIQ, Desipramine, and Their Combined Administration on the Concentration of Noradrenaline and Its Metabolites in Rat Brain Structures

In the frontal cortex, the two-way ANOVA revealed a significant effect of 1MeTIQ on the concentration of noradrenaline (*F*[1,19] = 13.9, *P* < 0.01) (Table 2). The statistical analysis demonstrated no effect of desipramine (*F*[1,19] = 0.04, N.S.) on the level of noradrenaline. At the same time, the interaction of 1MeTIQ and desipramine was significant (*F*[1,19] = 6.2, *P* < 0.05) (Table 2). The post hoc test showed that 1MeTIQ given alone as well as combined with desipramine produced an increase in the concentration of noradrenaline (by about 50 and 30 %, respectively). The two-way ANOVA indicated a significant effect of 1MeTIQ administration (*F*[1,19] = 46, *P* < 0.01) on the concentration of NMN in the frontal cortex (Table 2). At the same time, the effect of desipramine (*F*[1,19] = 0.97, N.S.) as well as of the interaction between 1MeTIQ and desipramine (*F*[1,19] = 0.01, N.S.) on the level of NMN were not significant (Table 2). The Duncan’s post hoc test showed that 1MeTIQ administered alone as well as combined with desipramine produced an increase in the concentration of NMN by about 200 % (*P* < 0.01).The two-way ANOVA indicated a significant effect of treatment with 1MeTIQ (*F*[1,19] = 6.36, *P* < 0.05) on the level of noradrenaline in the hypothalamus (Table 2). The same statistical test showed no effect of desipramine (*F*[1,19] = 0.58, N.S.) and no effect of interaction between 1MeTIQ versus desipramine (*F*[1,19] = 0.83, N.S.) in affecting noradrenaline concentration. The Duncan’s post hoc test demonstrated that 1MeTIQ given concomitantly with desipramine elevated the level of noradrenaline by about 15 % (*P* < 0.05) (Table 2). In the hypothalamus, the two-way ANOVA revealed a significant effect of 1MeTIQ (*F*[1,19] = 50, *P* < 0.01) and desipramine (*F*[1,19] = 32.1, *P* < 0.01) on NMN concentration. At the same time, the interaction between 1MeTIQ versus desipramine for the level of NMN was not significant (*F*[1,19] = 0.73, N.S.) (Table 2). The post hoc analysis showed that treatment with 1MeTIQ or desipramine produced elevation of NMN concentration by about 80 % (*P* < 0.01), while in the combination group, the level of NMN increased by about 250 % (*P* < 0.01) (Table 2).

**Table 2:** The influence of 1MeTIQ, desipramine, and combined administration on noradrenaline and its metabolite concentration in rat brain structures

Treatment 1	Treatment 2	NA (ng/g t)	NMN (ng/g t)
Frontal cortex			
Saline	Saline	209 ± 5	10 ± 1
	Desipramine 10	247 ± 23	13 ± 2
1MeTIQ10	Saline	298 ± 6**^#^	30 ± 1**^##^
	Desipramine 10	265 ± 6*	32 ± 4**^##^
Effect of 1MeTIQ		*F*_(1/19)_ = 13.9	*F*_(1/19)_ = 46
		*P* < 0.01	*P* < 0.01
Effect of desipramine		*F*_(1/19)_ = 0.04	*F*_(1/19)_ = 0.97
		N.S.	N.S.
Effect of interaction 1MeTIQ + desipramine		*F*_(1/19)_ = 6.2	*F*_(1/19)_ = 0.01
		*P* < 0.05	N.S.
Hypothalamus			
Saline	Saline	1525 ± 71	18 ± 1
	Desipramine 10	1512 ± 85	30 ± 2**
1MeTIQ 10	Saline	1664 ± 64	33 ± 2**
	Desipramine 10	1809 ± 81*^#^	49 ± 3**^##^
Effect of 1MeTIQ		*F*_(1/19)_ = 6.36	*F*_(1/19)_ = 50
		*P* < 0.05	*P* < 0.01
Effect of desipramine		*F*_(1/19)_ = 0.58	*F*_(1/19)_ = 32.1
		N.S.	*P* < 0.01
Effect of interaction 1MeTIQ + desipramine		*F*_(1/19)_ = 0.83	*F*_(1/19)_ = 0.73
		N.S.	N.S.
Striatum			
Saline	Saline	41 ± 4	13 ± 1
	Desipramine 10	44 ± 7	10 ± 1
1MeTIQ 10	Saline	11 ± 4**^##^	8 ± 1*
	Desipramine 10	57 ± 4	15 ± 1^#^
Effect of 1MeTIQ		*F*_(1/19)_ = 19.7	*F*_(1/19)_ = 4.69
		*P* < 0.01	*P* < 0.05
Effect of desipramine		*F*_(1/19)_ = 2.26	*F*_(1/19)_ = 0.04
		N.S.	N.S.
Effect of interaction 1MeTIQ + desipramine		*F*_(1/19)_ = 14.8	*F*_(1/19)_ = 18.72
		*P* < 0.01	*P* < 0.01

1MeTIQ was administered once in a dose 10 mg/kg i.p. Desipramine was administered in acute dose 10 mg/kg i.p. In the mixed group, 1MeTIQ was given 15 min before desipramine injection. Control group was treated with saline. The rats were decapitated about 1 h after desipramine administration, immediately after completion of a behavioral test (FST). The results are expressed as the mean ± SEM from 4 to 6 samples. The indices were calculated using concentrations from individual tissue samples. The concentration of noradrenaline and its metabolite was measured in ng/g tissue. The data were analyzed by means of two-way analysis of variance ANOVA, followed when appropriate, by Duncan’s post hoc test. Statistical significance: * *P* < 0.05, ** *P* < 0.01 versus Control group; ^#^ *P* < 0.05, ^##^ *P* < 0.01 versus desipramine-treated group

In the striatum, a two-way ANOVA indicated a significant effect of 1MeTIQ (*F*[1,19] = 19.7, *P* < 0.01) on the noradrenaline concentration. In opposite to this, the effect of desipramine was not significant (*F*[1,19] = 2.26, N.S.) (Table 2). The same statistical analysis demonstrated that the interaction between 1MeTIQ versus desipramine was significant (*F*[1,19] = 14.8, *P* < 0.01) (Table 2). The Duncan’s post hoc test showed that 1MeTIQ reduced the concentration of noradrenaline by about 70 % (*P* < 0.01). A two-way ANOVA revealed a significant effect of 1MeTIQ(*F*[1,19] = 4.69, *P* < 0.05) on NMN concentration in the rat striatum (Table 2). At the same time, the effect of desipramine was not significant (*F*[1,19] = 0.04, N.S.) but the interaction between 1MeTIQ versus desipramine was significant (*F*[1,19] = 18.72, *P* < 0.01) in respect of the level of NMN (Table 2).

#### The Effect of 1MeTIQ, Desipramine, and Their Combined Administration on the Rate of Noradrenaline Metabolism in the Rat Hypothalamus

**Fig. 4:**
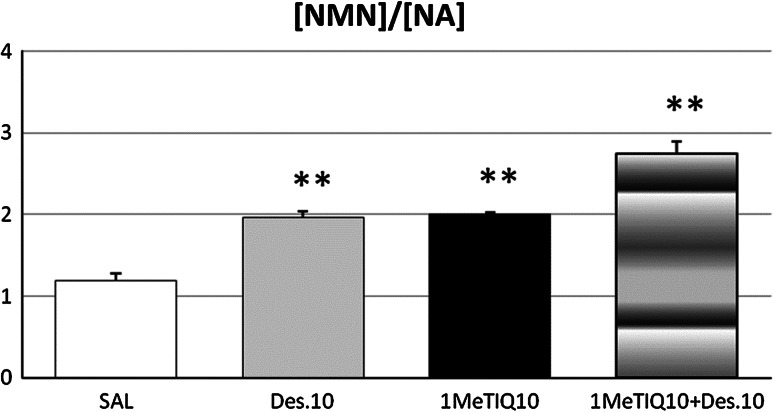
The effect of 1MeTIQ compared with desipramine as well as combined administration of both this drugs on the rate of release of noradrenaline expressed as ([NMN]/[NA]) × 100 in rat hypothalamus. The rats received a single injection of saline (control), desipramine (10 mg/kg i.p.) or 1MeTIQ (10 mg/kg i.p.). In the mixed group, 1MeTIQ was given 15 min before desipramine injection. The rats were decapitated about 1 h after desipramine administration, immediately after completion of a behavioral test (FST). The results are expressed as the mean ± SEM from 4 to 6 samples. The indices were calculated using concentrations from individual tissue samples. The data were analyzed by means of two-way analysis of variance ANOVA, followed when appropriate, by Duncan’s post hoc test. Statistical significance: * *P* < 0.05, ** *P* < 0.01 versus control group

In the hypothalamus, the two-way ANOVA revealed a significant effect of 1MeTIQ (*F*[1,19] = 38.5, *P* < 0.01) and desipramine (*F*[1,19] = 36.1, *P* < 0.01) on the rate of noradrenaline metabolism measured as the index [NMN]/[NA] (Fig. 4). In contrast to this, the interaction between 1MeTIQ versus desipramine was not significant (*F*[1,19] = 0.02, N.S.). The post hoc test showed that 1MeTIQ and desipramine given alone increased the rate of noradrenaline metabolism by about 80 %, but if both drugs were given in combination, the elevation was even stronger (by about 180 %) (Fig. 4).

#### The Influence of 1MeTIQ, Desipramine, and Their Combined Administration on the Concentration of Serotonin and Its Metabolites in the Rat Brain Structures

In the hypothalamus, the two-way ANOVA revealed a significant effect of 1MeTIQ (10 mg/kg i.p.) (*F*[1,19] = 58.3, *P* < 0.01) on serotonin concentration (Table 3). In opposite to this, the effect of desipramine (10 mg/kg i.p.) (*F*[1,19] = 0.09, N.S.) or the 1MeTIQ versus desipramine interaction was not significant (*F*[1,19] = 1.12, N.S.). The Duncan’s post hoc test demonstrated that 1MeTIQ produced a significant elevation of serotonin concentration (by about 25 %, *P* < 0.01) in both treatment groups in the hypothalamus (Table 3). The two-way ANOVA indicated a significant effect of 1MeTIQ (*F*[1,19] = 4.62, *P* < 0.05) on the level of 5-HIAA in the hypothalamus (Table 3). The same statistical analysis showed that the effect of desipramine (*F*[1,19] = 0.51, N.S.) or 1MeTIQ and desipramine interaction on the regulation of 5-HIAA concentration (*F*[1,19] = 0.07, N.S.) were not significant (Table 3).

**Table 3:** The influence of 1MeTIQ, desipramine, and combined administration on serotonin and its metabolite concentration in rat brain structures

Treatment 1	Treatment 2	5-HT (ng/g t)	5-HIAA (ng/g t)
Hypothalamus			
Saline	Saline	921 ± 27	530 ± 27
	Desipramine 10	943 ± 25	553 ± 18
1MeTIQ 10	Saline	1,172 ± 17**^##^	485 ± 16
	Desipramine 10	1,132 ± 26**^##^	496 ± 17
Effect of 1MeTIQ		*F*_(1/19)_ = 58.3	*F*_(1/19)_ = 4.62
		*P* < 0.01	*P* < 0.05
Effect of desipramine		*F*_(1/19)_ = 0.09	*F*_(1/19)_ = 0.51
		N.S.	N.S.
Effect of interaction 1MeTIQ + desipramine		*F*_(1/19)_ = 1.12	*F*_(1/19)_ = 0.07
		N.S.	N.S.
Striatum			
Saline	Saline	454 ± 19	534 ± 26
	Desipramine 10	487 ± 21	628 ± 35
1MeTIQ 10	Saline	393 ± 11*^##^	565 ± 9
	Desipramine 10	499 ± 13	516 ± 31^#^
Effect of 1MeTIQ		*F*_(1/19)_ = 12.26	*F*_(1/19)_ = 1.49
		*P* < 0.01	N.S.
Effect of desipramine		*F*_(1/19)_ = 1.54	*F*_(1/19)_ = 0.45
		N.S.	N.S.
Effect of interaction 1MeTIQ + desipramine		*F*_(1/19)_ = 3.5	*F*_(1/19)_ = 4.61
		N.S.	*P* < 0.05

1MeTIQ was administered once in a dose 10 mg/kg i.p. Desipramine was administered in acute dose 10 mg/kg i.p. In the mixed group, 1MeTIQ was given 15 min before desipramine injection. Control group was treated with saline. The rats were decapitated about 1 h after desipramine administration, immediately after completion of a behavioral test (FST). The results are expressed as the mean ± SEM from 4 to 6 samples. The indices were calculated using concentrations from individual tissue samples. The concentration of serotonin and its metabolite was measured in ng/g tissue. The data were analyzed by means of two-way analysis of variance ANOVA, followed when appropriate, by Duncan’s post hoc test. Statistical significance: * *P* < 0.05, ** *P* < 0.01 versus Control group; ^#^ *P* < 0.05, ^##^ *P* < 0.01 versus desipramine-treated group

In the striatum, a two-way ANOVA revealed a significant effect of 1MeTIQ (*F*[1,19] = 12.26, *P* < 0.01) on serotonin concentration but the effect of desipramine (*F*[1,19] = 1.54, N.S.) was not significant (Table 3). At the same time, the interaction between 1MeTIQ versus desipramine was not significant (*F*[1,19] = 3.5, N.S.). The statistical analysis demonstrated no effect of 1MeTIQ (*F*[1,19] = 1.49, N.S.) or desipramine (*F*[1,19] = 0.45, N.S.) on 5-HIAA concentration (Table 3). The two-way ANOVA indicated a significant effect of 1MeTIQ versus desipramine interaction (*F*[1,19] = 4.61, *P* < 0.05) on the level of 5-HIAA.

#### The Effect of 1MeTIQ, Desipramine, and Their Combined Administration on the Rate of Serotonin Metabolism in the Rat Hypothalamus

**Fig. 5:**
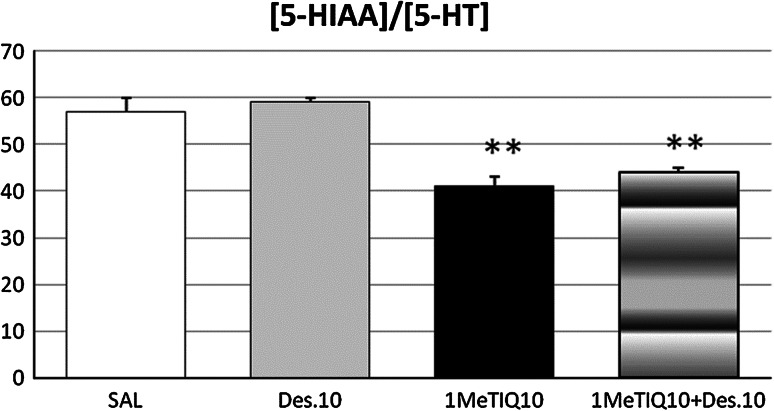
The effect of 1MeTIQ compared with desipramine as well as combined administration of both this drugs on the rate of final serotonin metabolism expressed as ([5-HIAA]/[5-HT]) × 100 in rat hypothalamus. The rats received a single injection of saline (control), desipramine (10 mg/kg i.p.) or 1MeTIQ (10 mg/kg i.p.). In the mixed group, 1MeTIQ was given 15 min before desipramine injection. The rats were decapitated about 1 h after desipramine administration, immediately after completion of a behavioral test (FST). The results are expressed as the mean ± SEM from 4 to 6 samples. The indices were calculated using concentrations from individual tissue samples. The data were analyzed by means of two-way analysis of variance ANOVA, followed when appropriate, by Duncan’s post hoc test. Statistical significance: * *P* < 0.05, ** *P* < 0.01 versus control group

The statistical analysis demonstrated a significant effect of 1MeTIQ (*F*[1,19] = 44.2, *P* < 0.01) on the rate of serotonin metabolism in the hypothalamus (Fig. 5). In opposite to this, the effect of desipramine (*F*[1,19] = 0.55, N.S.) or the interaction between 1MeTIQ and desipramine (*F*[1,19] = 0.09, N.S.) was not significant (Fig. 5). The Duncan’s post hoc test indicated that 1MeTIQ given alone and combined with desipramine reduced the serotonin metabolism rate (by about 30 %, *P* < 0.01).

## Discussion

The main finding of this paper is that the endogenous neuroprotective compound, 1MeTIQ administered systemically to rats produced antidepressant-like effect in the FST. This effect was intensified by combined administration of 1MeTIQ and desipramine, a classic tricyclic antidepressant. Neurochemical data showed that this effect was connected with the activation of all monoaminergic (dopaminergic, serotoninergic, and noradrenergic) systems.

Desipramine is a well-known tricyclic antidepressant which acts as a selective noradrenaline reuptake inhibitor (Grunewald et al. 1979; Javaid et al. 1979; Wroblewski et al. 1996). In rodents, desipramine induced a significant decrease in the immobility time in the forced swimming test (Redrobe et al. 1996; Lucki et al. 2001; Holmes 2003; Kim et al. 2010) with simultaneous elevation of the climbing time. The modified FST measures the frequency of different types of active behaviors: swimming, which is sensitive to serotoninergic compounds, such as SSRIs, and climbing, which is sensitive to tricyclic antidepressants and drugs with selective effects on catecholamine transmission (Cryan and Lucki 2000; Cryan et al. 2005; Detke et al. 1995). As shown by Detke et al. (1995), the increase in climbing activity is connected with an enhanced noradrenergic system activation.

Our present data from the behavioral experiments demonstrated that acute systemic administration of 1MeTIQ in a low dose (10 mg/kg i.p.) produced antidepressant-like effect by reducing the immobility time (Fig. 1a) with simultaneous elevation of the climbing time (Fig. 1c). This effect was similar to the influence induced by desipramine given in a lower dose (10 mg/kg i.p.) (Fig. 1a, c). The higher dose of desipramine produced a stronger decrease in the immobility time (Fig. 1a) with simultaneous powerful elevation of the climbing time (Fig. 1c). In the combination group, when 1MeTIQ was given together with a lower dose of desipramine, the reduction of the immobility time was slightly stronger than in the 1MeTIQ group with a significant increase in the swimming time (Fig. 1a, b). These data coming from the behavioral FST suggest that 1MeTIQ (given alone in a low dose), similarly to desipramine, influences mainly the catecholaminergic system. In opposite to this, when 1MeTIQ was administered in combination with desipramine, the activation of serotoninergic system seemed to be the dominant which was reflected by the swimming behavior in this group (Fig. 1b).

In our earlier studies, we demonstrated that 1MeTIQ was a reversible, short-acting and moderate inhibitor of both MAO-A and MAO-B activity (Antkiewicz-Michaluk et al. 2001; Patsenka and Antkiewicz-Michaluk 2004). There are evidences that MAO inhibitors have several particularly useful therapeutic properties, such as the blockade of the oxidation pathway (leading to reduced free radical production) in monoamine catabolism (Youdim et al. 2006) and the increase in the concentration of monoamines in the brain by inhibition of their catabolism (Alvarez et al. 1999; Haefely et al. 1992). This mechanism of action is responsible not only for neuroprotective effects but also for antidepressant-like activity of MAO inhibitors (e.g., selegiline or moclobemide) in different animal models (Cryan et al. 2005; Kitamura et al. 2008; Miura et al. 1996; Youdim and Bakhle 2006). In order to exclude the psychostimulating activity of 1MeTIQ, we have examined the influence of its administration (alone and combined with desipramine) on locomotor activity of rats. 1MeTIQ administered in a dose of 10 mg/kg i.p. or desipramine (10 mg/kg i.p.) induced a significant decrease in locomotor activity (Fig. 2). An even stronger reducing effect on motility was observed in the combination group (Fig. 2). These results clearly show that 1MeTIQ, like desipramine, did not have psychostimulant properties and the prolonged climbing or swimming time (in the combination group) in the forced swimming test was the result of an increased motivation to escape from the stressful situation, i.e., placing the animal in the cylinder with water.

The results from behavioral experiments were confirmed by the neurochemical data. For presentation the neurochemical results we selected two brain structures: connected with monoaminergic function: the striatum—as a representative of the dopaminergic system and motor function, and the hypothalamus for noradrenergic system. It should be borne in mind that serotonin nerve endings are present in large numbers in both analyzed brain structures. Some authors demonstrated that striatal dopamine play an important role in the pathophysiology of depression by modulating emotional and motor symptoms (Byrum et al. 1999; Newberg et al. 2007; Rogers et al. 1998). Additionally, Amsterdam et al. (2012) suggested that greater striatal dopamine transporter density may represent a putative biomarker of depression. Also Shah et al. (1997) reported a decrease in dopamine function in the striatum of patients with major depression. Moreover, evidence from animal and human studies showed that stressful life events and the consequent hypothalamic–pituitary–adrenal (HPA) axis hyperactivity are among the most potent factors of depressive episodes (Swaab et al. 2005; Wang et al. 2010). As demonstrated in Table 1, 1MeTIQ in contrast to desipramine, significantly affected the dopaminergic system. It produced a significant elevation (*P* < 0.05) of the dopamine concentration in the hypothalamus. Additionally, 1MeTIQ induced a significant reduction (*P* < 0.01) in the level of the interneuronal dopamine metabolite, DOPAC with simultaneous elevation (*P* < 0.01) of the concentration of extraneuronal dopamine metabolite, 3-MT (Table 1). At the same time, the rate of dopamine oxidation expressed as the [DOPAC]/[DA] ratio was significantly (*P* < 0.01) decreased (Fig. 3a), and the marker of dopamine reuptake inhibition, i.e., the [3-MT]/[DOPAC] ratio was strongly increased (*P* < 0.01) (Fig. 3b). This is a very important property of 1MeTIQ because it not only induced elevation of the dopamine concentration in the synaptic cleft but also by blocking the activity of MAO it caused the reduction of free radical production in the dopaminergic neurons (Antkiewicz-Michaluk et al. 2001; Patsenka and Antkiewicz-Michaluk 2004). 1MeTIQ, as a moderate and reversible inhibitor of MAO-A and MAO-B (Patsenka and Antkiewicz-Michaluk 2004) seems to be safe and effective in clinic what can be important from practical point of view. The formation of free radicals leads to oxidative stress. Oxidative stress is a universal mechanism of inducing cell death (Dykens 1999). Maes et al. (2009) postulated a new theory of depression, which was called “the inflammatory and neurodegenerative hypothesis of depression” which suggests that the activation of inflammatory and oxidative stress pathways is the cause of development of depression (Maes et al. 2008). 1MeTIQ given concomitantly with desipramine produced a significant elevation (*P* < 0.01) of the concentration of dopamine with simultaneous reduction of the DOPAC level (*P* < 0.05) (Table 1). In the combination group, we observed a significant decrease in the rate of dopamine oxidation pathway expressed as the [DOPAC]/[DA] ratio of the magnitude similar to the 1MeTIQ group (Fig. 3a), however, the marker of dopamine reuptake inhibition; i.e., the [3-MT]/[DOPAC] ratio elevated by 1MeTIQ returned to control values in the combined treatment group (Fig. 3b). Simultaneously, the rate of total dopamine metabolism measured as the [HVA]/[DA] × 100 ratio was significantly (*P* < 0.05) decreased (Fig. 3c).

Similarly to desipramine, 1MeTIQ did not influence the level of noradrenaline but induced a significant (*P* < 0.01) elevation of the NMN concentration in the hypothalamus (Table 2). As shown in Fig. 4, 1MeTIQ produced an increase (*P* < 0.01) in the rate of noradrenaline release measured as [NMN]/[NA] × 100 with the same effectiveness like desipramine. However, in the frontal cortex, 1MeTIQ acted more strongly than desipramine and significantly (*P* < 0.01) increased the concentration of both, noradrenaline and NMN (Table 2). This effects of 1MeTIQ may be associated with its ability to inhibit the noradrenaline reuptake process. Noradrenaline released into the synaptic cleft is catabolized by COMT to NMN by the process of methylation. The increased amount of NMN indicates the elevated release of noradrenaline or the inhibition its uptake into the neuron. 1MeTIQ given in combination with desipramine produced a significant increase in the concentration of noradrenaline (*P* < 0.05) and its direct metabolite, NMN (*P* < 0.01) (Table 2). At the same time, the rate of extraneuronal noradrenaline catabolism expressed as [NMN]/[NA] × 100 was strongly elevated (more than in the groups treated with each drug alone) (Fig. 4). The strongest activation of noradrenergic system was observed in the combined treatment group in the hypothalamus where both, the concentration of noradrenaline and its metabolite, NMN was significantly elevated (Table 2).

The present data demonstrated that desipramine given in a dose (10 mg/kg i.p.) did not affect the concentration of serotonin and its metabolite, 5-HIAA (Table 3). Also the rate of serotonin metabolism measured as [5-HIAA]/[5-HT] × 100 was not changed by desipramine (Fig. 5). In contrast to desipramine, 1MeTIQ produced a significant elevation (*P* < 0.01) of the serotonin concentration in the hypothalamus (Table 3). Additionally, the rate of serotonin metabolism estimated as [5-HIAA]/[5-HT] × 100 was significantly (*P* < 0.01) decreased in this group (Fig. 5). The observed neurochemical changes in the serotoninergic system are the result of the inhibition of MAO activity in the rat brain by 1MeTIQ (Patsenka et al. 2004). The neurochemical data demonstrated that in the hypothalamus, the concentration of serotonin was significantly increased (*P* < 0.01) in the combined treatment group (Table 3). Simultaneously, the rate of serotonin metabolism measured as [5-HIAA]/[5-HT] × 100 was significantly decreased (*P* < 0.01) in this group (Fig. 5).

Our present data demonstrate that 1MeTIQ is characterized by a wide spectrum of actions on all monoaminergic systems in the rat brain. Thanks to its ability to inhibit both MAO-A and MAO-B activity (Patsenka and Antkiewicz-Michaluk 2004) and to scavenge free radicals (Antkiewicz-Michaluk et al. 2006); 1MeTIQ may be useful not only for the therapy of neurodegenerative disease but also in the treatment of depression.

## Conclusion

In summary, we have found that 1MeTIQ an endogenous neuroprotective compound, given alone and in combination with desipramine, exhibited antidepressant-like activity in the forced swimming test in the rat. The neurochemical results presented in this paper strongly support this suggestion. Antidepressant-like properties of 1MeTIQ are associated with the activation of all monoaminergic systems in the brain mainly by MAO inhibition and free radical scavenging properties. Owning to these mechanisms of action, 1MeTIQ may be very beneficial from the clinical point of view as a new antidepressant.
